# When nasal cytology detects acute lymphoblastic leukaemia: New diagnostical implications

**DOI:** 10.1111/cyt.13109

**Published:** 2022-02-14

**Authors:** Matteo Gelardi, Rossana Giancaspro, Pietro Pecoraro, Michele Cassano

**Affiliations:** ^1^ Department of Otolaryngology University Hospital of Foggia Foggia Italy; ^2^ ENT Specialist ASP 6 Palermo Italy

## Abstract

Nasal cytology is a diagnostic tool that evaluates the normal and pathological aspects of the nasal mucosa, by identifying and counting the cell types and their morphology. Since the cytological characteristics of the infiltrating inflammatory cells could reflect any cytopathological alterations of the blood circulating cells, it is not surprising that pathological findings of patients affected by oncohaematological diseases could also be found in nasal cytology. Therefore, this diagnostic tool could be applied to other branches of medicine including haematology.

## 1 CASE HISTORY

A previously healthy non‐smoking 42‐year‐old man came to our attention complaining of nasal hyperactivity, nasal obstruction, and anosmia for about 3 months. The patient had already undergone an allergological evaluation and skin‐prick tests (SPTs), which were negative for common allergens. Anterior rhinoscopy and nasal endoscopic examination revealed only a slight irregularity of the nasopharyngeal mucosa. The otoscopic exam revealed right catarrhal otitis. The oropharynx examination and cervical palpation were mostly normal. We performed nasal cytology, collecting the samples by Nasal Scraping®, under anterior rhinoscopy, from the middle part of the inferior turbinates.

**FIGURE 1:**
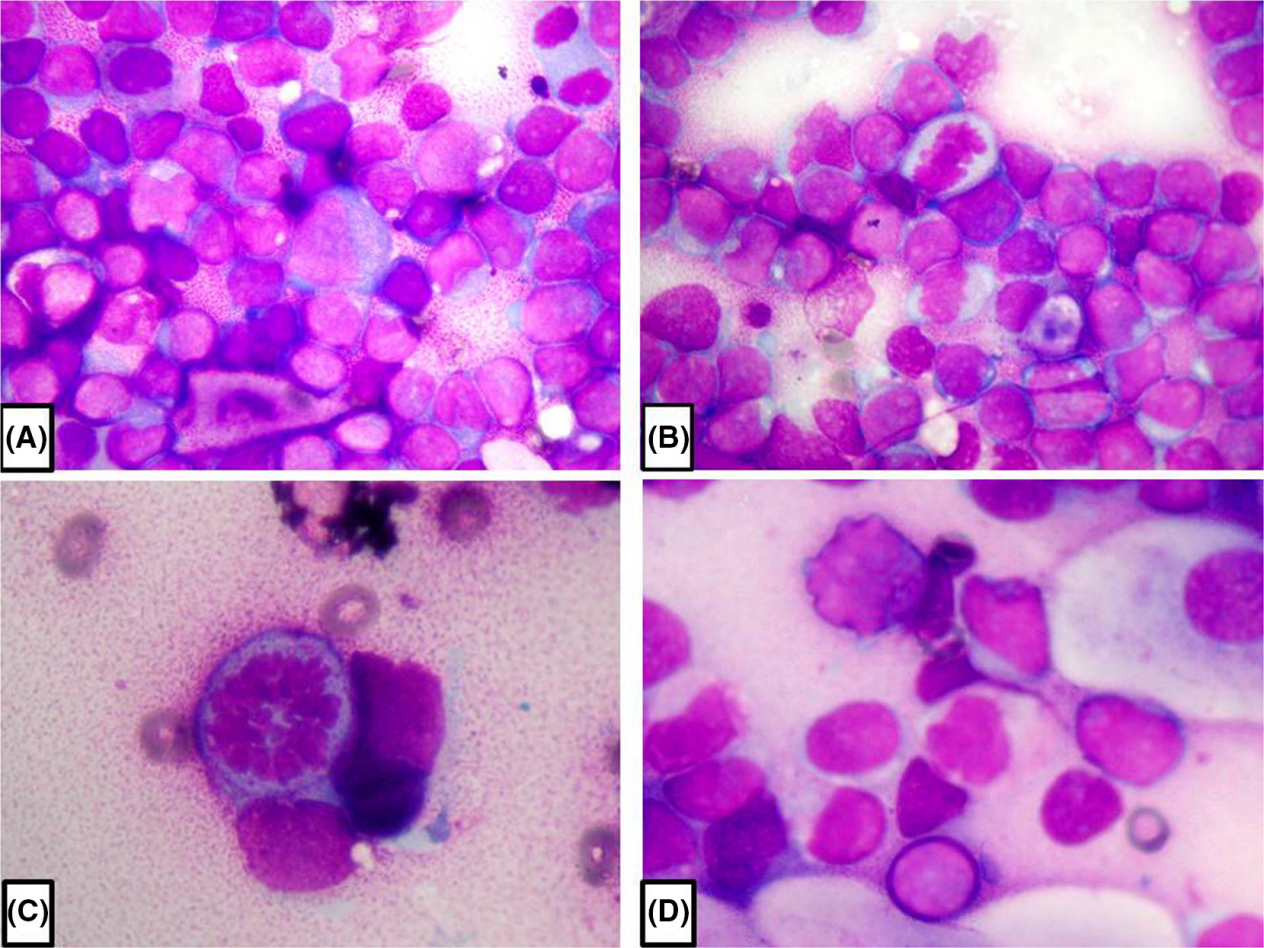
Nasal cytology. Massive lymphocytic infiltrate (A,B) characterised by abnormal cytopathological features, including irregular nuclei, numerous mitotic figures (B,C), dyschromic chromatin and compacted chromatin at the periphery of the nuclei (D). May‐Grünwald‐Giemsa staining. Magnification: ×1000 (A,B); ×1000 with a camera magnification factor of 2.2× (C, D)

Nasal cytology revealed a massive lymphocytic infiltrate, characterised by abnormal cytopathological features, including irregular nuclei, dyschromic chromatin and compacted chromatin at the periphery of the nuclei, and numerous mitotic figures (Figure 1A‐D). Subsequently, the patient underwent laboratory blood tests, neck ultrasonography (US) examination, computerised tomography (CT) scan and a magnetic resonance imaging (MRI) of the facial mass with and without contrast medium, blood smear, and nasopharyngeal biopsy, in light of the previously detected slight mucosal irregularity. Haematochemical examination revealed leucocytosis (WBC =42.65 10³/µl) with inverted formula (neutrophils 6.4%, lymphocytes 67.6%, monocytes 25.8%). Neck US showed bilateral laterocervical lymphadenopathy. The CT and MRI investigations only showed mucous retention cysts in the left maxillary sinus.

## 2 MORPHOLOGY QUIZ

1. What is the most probable cytological diagnosis based on the clinical history, nasal cytology, blood exams, and radiological examinations?
  - Allergic rhinitis
  - Non‐allergic rhinitis
  - Acute lymphoblastic leukaemia
  - Viral lymphocytosis
2. Which are the main cellular features shown in Figure 1?
  - Giant cells, multinucleated or with bilobed nuclei, prominent eosinophilic inclusion‐like nucleoli (Reed‐Stenberg cells)
  - Irregular nuclei, numerous mitotic figures, compacted dyschromic chromatin at the periphery of the nuclei
  - No cytological alterations
  - Macrophages with relatively clear cytoplasm, starry‐sky appearance
3. Which stain is used for nasal cytology?
  - May‐Grünwald‐Giemsa
  - Haematoxylin‐Eosin
  - Mallory‐Azan
  - Nissl
4. Which procedure is considered diagnostic for acute lymphoblastic leukaemia?
  - Lumbar puncture
  - Lymph node biopsy
  - Bone marrow biopsy
  - CT scans

## 3 DISCUSSION

Nasal cytology is a widely recognised and easy‐to‐apply diagnostic procedure that allows assessing the normal and pathological aspects of the nasal mucosa, by identifying and counting the cell types and their morphology.^1^ Since the cytological characteristics of the infiltrating inflammatory cells could reflect any cytopathological alterations of the blood circulating cells, it is not surprising that pathological findings of patients affected by oncohaematological diseases could also be found in nasal cytology. However, to the best of our knowledge, no studies have been reported regarding nasal cytology findings of patients with diseases.

We report the first case of acute lymphoblastic leukaemia (ALL) whose diagnostic suspicion was posed thanks to nasal cytology, and subsequently confirmed by oncohaematological examinations.

During inflammation, leucocytes circulating in the blood stream exit the vasculature in a process called leucocyte transendothelial migration, which consists of well‐established steps, including rolling, adhesion, crawling, diapedesis, and sub‐endothelial crawling, and migrate into the tissues through a process of chemotaxis.^2^ It is therefore deductible that if the circulating leucocytes show cytopathological alterations, the same alterations will be found in the leucocytes infiltrating tissues.

Our patient complained of signs and symptoms of a vasomotor rhinitis, and given the negative SPTs, we expected to find an inflammatory infiltrate pathognomonic of a vasomotor non‐allergic rhinitis on nasal cytology. However, the cytological findings were unexpected, not only for the presence of a conspicuous lymphocytic infiltrate, but also for the presence of several cytopathological alterations and numerous mitotic figures. Such a conspicuous lymphocyte infiltrate, almost completely occupying the fields, had never been described before as a nasal cytology finding. Furthermore, the cytopathological alterations and the elevated mitotic index led us to suspect a lymphoproliferative process. In particular, our patient was diagnosed with ALL, which has a high prevalence rate in children and young adults. Fever, fatigue, dizziness, dyspnoea, bleeding manifestations, lymphadenopathy, coagulopathy, increased susceptibility to infections, and hepatosplenomegaly are the most common symptoms. ALL arises from the uncontrolled proliferation of lymphoid precursor cells and the diagnosis requires 20% or more blast cells in bone marrow aspirate and biopsy materials. Moreover, a correct diagnostic framework requires a broad spectrum of information derived from several investigations, such as cell phenotyping, cytochemistry, cytogenetics, and molecular genetics. Despite technological and diagnostic advances, cellular morphology remains the frontline haematological diagnostic technique, since the observation of blasts and cytopathological alterations in the peripheral blood smears raise the first suspicion of leukaemia.^3^ Due to the observation of similar cytopathological alterations in the cells of the nasal inflammatory infiltrate, we suspected an ongoing oncohaematological process. Actually, the involvement of the nose or the paranasal sinuses in patients suffering from leukaemia is a rather rare event. Moreover, extramedullary leukaemic infiltration typically manifests as soft tissue masses, which can be the initial presentation of a haematopoietic malignancy.^4^ The patient in question did not present appreciable masses either on physical examination or radiological investigations. Probably the conspicuous nasal lymphocytic infiltrate was caused by the vasomotor rhinitis from which the patient suffered. However, due to the ongoing oncohaematological process, infiltrating cells showed the typical leukaemic characteristics.

To date, nasal cytology is considered an easy and repeatable tool which allows correctly framing the rhino‐allergologic patient and monitoring the effectiveness of any treatments over time. However, the applications of this diagnostic tool could potentially be extended to other branches of medicine, including haematology. In this context, further studies are needed in order to understand the possible role of nasal cytology in the early diagnosis and follow‐up of oncohaematological diseases.

**ANSWERS TO MORPHOLOGY QUIZ**

1. c
2. b
3. a
4. c

## INFORMED CONSENT

Written informed consent was obtained from the patient for publication of this article and any accompanying images.

## Data Availability

Data available on request from the authors.

## References

[1] Gelardi M , Giancaspro R , Pecoraro P , Cassano M . Nasal cytology in allergic rhinitis: rare observation of pollen degranulation. Int Forum Allergy Rhinol. 2021;11(12):1710‐1711. doi:10.1002/alr.22860 34185966

[2] Grönloh ML , Arts JJ , van Buul JD . Neutrophil transendothelial migration hotspots – mechanisms and implications. J Cell Sci. 2021;134: jcs255653. 33795378 10.1242/jcs.255653

[3] Herráez‐Aguilar D , Madrazo E , López‐Menéndez H , Ramírez M , Monroy F , Redondo‐Muñoz J . Multiple particle tracking analysis in isolated nuclei reveals the mechanical phenotype of leukemia cells. Sci Rep. 2020;10:6707. 32317728 10.1038/s41598-020-63682-5 PMC7174401

[4] Wang YM , Mo JQ , Kuo DJ , Wong V . MLL rearranged acute lymphoblastic leukaemia presenting as a maxillary sinus mass with a discordant immunophenotypic profile from the bone marrow. BMJ Case Rep. 2019;12:e227400. 10.1136/bcr-2018-227400 PMC6388888 30772833

